# In Silico/In Vitro Strategies Leading to the Discovery of New Nonribosomal Peptide and Polyketide Antibiotics Active against Human Pathogens

**DOI:** 10.3390/microorganisms9112297

**Published:** 2021-11-05

**Authors:** Sami Khabthani, Jean-Marc Rolain, Vicky Merhej

**Affiliations:** 1 Aix Marseille Université, IRD, APHM, MEPHI, 27 Boulevard Jean Moulin, CEDEX 05, 13385 Marseille, France; samikhabthani@outlook.fr (S.K.); jean-marc.rolain@univ-amu.fr (J.-M.R.); 2 IHU Méditerranée Infection, 19-21 Boulevard Jean Moulin, CEDEX 05, 13385 Marseille, France

**Keywords:** new NRP-PK antibiotics, in silico/in vitro strategies, human pathogens, bottom-up approach

## Abstract

Antibiotics are majorly important molecules for human health. Following the golden age of antibiotic discovery, a period of decline ensued, characterised by the rediscovery of the same molecules. At the same time, new culture techniques and high-throughput sequencing enabled the discovery of new microorganisms that represent a potential source of interesting new antimicrobial substances to explore. The aim of this review is to present recently discovered nonribosomal peptide (NRP) and polyketide (PK) molecules with antimicrobial activity against human pathogens. We highlight the different in silico/in vitro strategies and approaches that led to their discovery. As a result of technological progress and a better understanding of the NRP and PK synthesis mechanisms, these new antibiotic compounds provide an additional option in human medical treatment and a potential way out of the impasse of antibiotic resistance.

## 1. Introduction and Historical Context

Antimicrobials are probably one of the most successful forms of medical treatments in the history of medicine. Their discovery is due both to chance and to the ingenuity of Alexander Fleming, who noticed that *Penicillium notatum* inhibited the growth of *Staphylococcus aureus* colonies. This observation gave rise to the antibiotic era through the discovery of penicillin. Penicillin was purified and isolated, but not industrialised at this point [1]. Industrial production came over 10 years later, led by Howard Florey and Ernst Boris Chain, using *Penicillium chrysogenum* [2]. René Dubos, a French microbiologist, believed in the principle of the soil providing natural active substances against pathogenic bacteria. This was based on his experiments on “antibiosis”. In 1939, Dubos discovered gramicidin, the first clinically used antibiotic [3]. This experimental design inspired researchers to develop new methods to discover antibiotics. Waksman established a platform that consists of screening mainly soil-derived bacteria, particularly *Actinomycetes* [4], and identified actinomycin and streptomycin. These efforts opened the door to the “golden age of antibiotic discovery” [5]. Pharmaceutical companies have continued to apply these approaches to extract and purify most of the antibiotics used today, such as erythromycin, tetracycline, vancomycin, rifamycin, and others [6]. In the 1960s, the rate of discovery of new antibiotics dropped sharply due to the high rate of rediscovery and the difficulties of characterising unknown compounds. This allowed the pharmaceutical companies to turn away from this type of research [7,8]. In the past fifty decades, only two new classes of antibiotics have been discovered. With this, international organisations have noticed a serious problem, now considering antimicrobial resistance as a major public health problem [9].

The use of natural antimicrobial compounds in human treatment is a great example of a diversion of developing resources from microbes. These secondary metabolites have been reengineered to be used by humans in order to combat several infectious diseases. They had a positive impact on human health, and they helped to stem the scourge of several diseases. Fundamentally, however, bacteria use these compounds for their self-defence. They evolve in complex ecosystems in which they are continuously in “war with one another” to ensure their own survival. To this end, and coupled with other strategies, they release antimicrobial substances into the environment. Then, they consequently contribute to the regulation of the populations of other bacterial populations with which they compete [10,11]. A number of marketed antibiotics are nonribosomal peptides (NRP) and polyketides (PK), which have been extracted from microbes in culture media, and have sometimes been modified to have a better efficiency or to reduce toxicity. It should be noted that the total pharmaceutical synthesis of these compounds is severely limited by the singularity and specificity of their ribosomally independent natural synthesis process. NRP and PK are synthetised on large nonribosomal peptide synthetase (NRPS) and polyketide synthase (PKS) enzyme complexes, respectively. These enzymes are modular enzymes that function in an assembly line fashion [12,13]. These mega-enzymes are encoded in the bacterial genome by biosynthetic gene cluster (BGCs) [14].

Because of the antimicrobial properties of NRPS and PKS products, much effort has gone in to the exploration of novel NRP and PK, with the aim of developing new approaches to fight the emerging resistance profile of pathogenic bacteria. With the improvement of new sequencing technologies and bioinformatic software [15], genome mining is becoming a key strategy to discover new antibiotics. This is due to its ability to easily screen for interesting bacterial genomes and metagenomes at a constantly decreasing cost and with better efficiency. In this review, we wanted to catalogue recently discovered new NRP-PK antibiotics and describe different in silico/in vitro strategies that made their discovery possible.

## 2. Overview of Polyketide Synthase (PKS) and Nonribosomal Peptide Synthase (NRPS)

NRP and PK are two diverse families with a broad variety of complex chemical structures and pharmacological activities [13]. A large proportion of the antibiotics used in human medicine belong to the NRP and PK classes, and they are well known in infectiology, for example penicillin, vancomycin, daptomycin, erythromycin, mupirocin, and oxytetracycline (Table 1). Since the first observations were made by the chemist Jamie Collie at the University of London in 1893, establishing the structure of orcinol, to the theory of Robert Robinson in 1955 and Birch’s work, many attempts have been made to characterise the biosynthetic pathways of these secondary metabolites [12]. The multienzymatic thiotemplate model was retained as a plausible explanation, and a growing number of enzymatic domains have been identified. These domains are involved in a variety of reactions necessary for the basic assembly line system.

**Figure 1:**
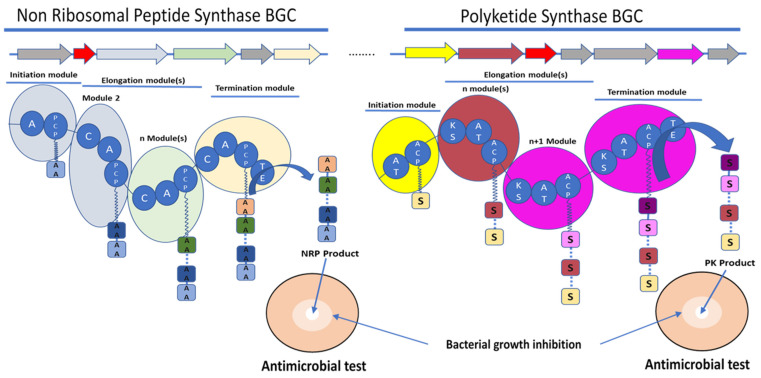
Schematic representation of the biosynthetic gene clusters (BGC) (arrows) and their encoded synthetase assembly lines (similarly coloured bubbles) involved in the biosynthesis of non-ribosomal peptide (NRP) and polyketide (PK) molecules. Non-ribosomal peptide synthase (NRPS) core domains are labelled as follow: A—adenylation domain, C—condensation domain, and PCP—peptidyl carrier protein. Polyketide synthase (PKS) core domains are labelled as follow: AT—acetylation domain, KS—ketosynthase, and ACP—acyl carrier protein. TE—thioesterase was a common domain to NRPS and PKS. These domains have various functions in the synthesis of the final molecule. A and AT domains are involved in the selection and activation of the substrate, C and KS domains are involved in the condensation of the substrate AA for amino acid or S for acyl-CoA or malonyl-CoA with the growing NRP or PK, respectively. ACP and PCP play a role in the transfer of the substrate between the different modules. TE releases the final molecule. The red arrows rather encode for immunity or resistance genes to the synthesized antibiotic.

Inspired by the study of the biosynthesis of actinorhodin, [16] researchers identified the erythromycin BGC using different strategies, including sequencing adjacent parts of the gene coding for erythromycin resistance [17], looking for sequences similar to fatty acid sequence and other PKS enzymes [17], or mutated genes involved in the synthesis of 6-deoxyerythronolide B (6-dEB) [18,19]. Erythromycin polyketide synthase is encoded by three genes, *eryAI, eryAII*, and *eryAIII*, which code for three multienzymatic polypeptide 6-deoxyerythronolide B synthases, DEBS1, DEBS2, and DEBS3, respectively. Each of these giant proteins contain domains or catalytic sites [12]. Erythromycin is synthetised according to the biosynthesis mode of type I PKS. Type I PKS is a multifunctional enzyme organised into several modules (Figure 1). Each module contains three core domains necessary for the definition of type 1 PKS, namely acyl transferase (AT), ketosynthase (KS), and acyl carrier protein (ACP). The biosynthesis mode of type I PKS is linear. An acyl-coenzyme A is used as substrate and is selected by the AT. ACP, then, transfers the acyl-coenzyme A into the next module, and KS catalyses a Claisen condensation between acyl-coenzyme A and the growing polyketide chain attached to the ACP domain (Figure 1). Recent studies have questioned the definition of modules in polyketide synthase based on evolutionary analysis [20]. The authors show that domains that migrate together over the course of evolution of PKS assembly lines do not correspond to the known definitions of modules [21]. Two other types of PKS biosynthesis are known: type II and type III PKS [22]. The type II PKS is iterative; it is composed of two core domains: heterodimeric KS (KS and chain length factor subunits) and an ACP. Type II PKS usually acts by loading an α-carboxylated precursor onto an ACP, which is transferred to the active site of a KS for Claisen condensation and iterative lengthening using malonyl-coenzyme A (CoA) to result in a poly-β-keto chain [23]. This enzymatic machinery is responsible for the synthesis of aromatic polyketide such as tetracycline. Type III PKS are ACP independent and iterative homodimeric enzymes, composed of a single domain KS that plays the main role in the biosynthesis of natural products [24]. Type III PKSs are structurally less complex than type I or II PKSs and at least 15 families of type III PKSs have been identified to date [25].

The ribosome-independent synthesis mechanism of NRPS was discovered by studying the biosynthesis of unusual peptides in the *Bacillus* species [13]. NRPS are multienzyme machineries that assemble peptides with diverse structures and functionalities [26]. Therefore, the amino acid substrate is activated by adenylation and thioesterification at specific reactive thiol groups, the peripheral cysteines of the multienzyme, on the same model of the fatty acid synthase. An intrinsic 4′-phosphopantetheine carrier was considered to interact with the thioesterified amino acids, ensuring a step-by-step elongation of the peptide product in a series of transpeptidation and transthiolation reactions. Therefore, NRPS are organised into distinct modular sections, each composed of catalytic domains with different functions. Three domains are considered as core and are essential for peptide elongation: adenylation (A) domains, peptidyl carrier protein or thiolation (PCP or T) domains, and condensation (C) domains [27]. The A domain is involved in the selection and activation of amino acid substrates with adenosine-5′-triphosphate (ATP), and it ligates it to the adjacent PCP domain to be incorporated into the NRP [28]. A domains are, therefore, involved in the large diversity of the NRP structure by selecting not only the 20 standard proteinogenic amino acids but also nonproteinogenic amino acids [13,29]. A domains own a specific code composed of 10 amino acids, which are responsible for substrate binding [30]. Some bioinformatics tools are able to predict the substrate to be selected by an amino acid by A domains and, therefore, the NRP amino acid composition [31,32,33]. C domains catalyse amide bond formation between amino acids and the growing peptide; they have a conserved HHxxxDG motif. This conserved motif is essential for the peptide bond formation [34]. Bioinformatic analyses revealed subclasses that are classified depending on the condensation between acceptor and donor and noncanonical members of the C superfamily [35]. The PCP domains transport the substrate between the different NRPS modules [35]. PCP domains must be post-translationally modified by the addition of the 4′-phosphopantetheine (PPT) to a conserved serin residue of the conserved GGXS motif [35]. The PCP domain does not play an active role in catalysis, but it has a central role in NRPS function. The PCP interacts with the A domain and other catalytic domains involved in the biosynthesis of NRP (Figure 1) [36].

## 3. The First Culture-Dependant Discoveries of Cultivable and Uncultivable Micro-Organisms

Since the discovery of the first antibiotics in 1928, finding new antibiotics has been dependent on culture results. The predicted producer microorganism is placed into microbial coculture with the target bacteria, which induces the production of compounds with antimicrobial activity. This method, known as the “top-down” approach by Luo et al. (2014) [37] led to the discovery of many antibiotics. The advantage of this method lies in the ease of use and the inexpensive materials required to prove that a microorganism has an antimicrobial effect. In a recent illustration of the efficiency of this method, Zipperer et al. (2016) constructed a nasal *Staphylococcus* collection and tested them for an antimicrobial activity by culture against commensal bacteria and opportunistic pathogens [38]. A specific strain of *Staphylococcus lugdunensis* has been shown to inhibit the growth of a nasal commensal *S. aureus*, vancomycin-resistant *Enterococcus* (VRE), glycopeptide-intermediate-resistant *S. aureus* (GISA), and methicillin-resistant *Staphylococcus aureus* (MRSA). The BGC responsible for the synthesis of the compound was found to be an NRPS and was revealed and characterised using transposon mutagenesis and bioinformatic analysis. This NRP antibiotic, named lugdunin, showed an interesting antimicrobial property on animal skin infection models.

Observations of the ecological niche can help us to make assumptions about the interbacterial competition, which can guide research into the underlying mechanisms of antagonism. Thus, epidemiological studies have shown that *S. lugdunensis*-positive patients are significantly less colonised by *S. aureus* than noncarrier *S. lugdunensis* patients [39]. This ecology-guided approach, combined with culture methods, is, therefore, highly interesting when it comes to studying the natural product that may be involved in the differences between the compositions of two similar environments. The discovery of teixobactin would not have been possible without the efforts made to develop the culture of the uncultivable bacteria [40]. Nichols et al. used an iChip [41], a multichannel device, to grow and isolate an uncultured bacteria from a sample of soil. From 10,000 isolates, a new species never previously cultured, called *Eleftheria terrae*, was tested against *S. aureus* and exhibited interesting antimicrobial activity. This Gram-negative bacterium was related to *Aquabacteria*, which were not known as antibiotic producers. Teixobactin is a NRP antibiotic encoded by two NRPS coding genes that were identified by a homology search tool. The amino acid composition of the NRP and the biosynthetic pathway were predicted in silico. Teixobactin exhibits an excellent antimicrobial activity against drug-resistant Gram-positive bacteria and *Mycobacterium tuberculosis*, and resistance to it has not yet been detected. This promising molecule is an example of how intensifying research to improve culture processes holds the potential to discover new bacterial species and genus that could be a source of new antibiotics (Table 2).

With the exception of the few examples of new antibiotics discovered using the culture-dependant approach, there are numerous drawbacks, with the fact that it is also tedious, time consuming, and sometimes, nonreproducible. As long as the search for antibiotics relied on the culture-dependant approach, there would be a high rate of rediscovery of already known compounds, which led to a reduction in the use of this approach in favour of other methods.

## 4. New Discoveries Allowed by Genome-Mining Approaches

**Figure 2:**
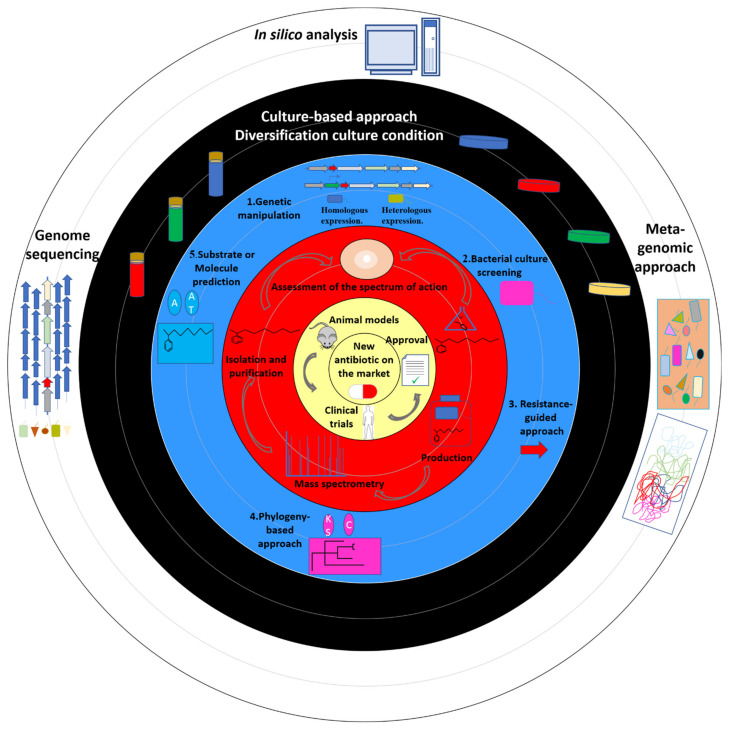
From materials and methods to market: schematic representation of materials and different in silico/in vitro strategies to identify new compounds with antimicrobial activity. The circles represent the different stages of the process.

Genome mining is a revolutionary approach to search for natural products synthesised by microorganisms, especially since high-throughput sequencing has become more accessible, and several pieces of bioinformatic software have become more and more powerful (Figure 2, Table 2). Many websites and web portals, including the antibiotics and secondary metabolite analysis shell (antiSMASH) [42], the nonribosomal polyketide urmite (NRPPUR) database [43], the secondary metabolite unknown regions finder (SMURF) [44], the natural product domain seeker (NAPDOS) [45], antibiotic-resistant target seeker (ARTS) [46], and others were developed to identify and characterise NRP and PK in microbial genomes. They contain databases and tools to identify the secondary metabolites, mainly using BLAST and HMMer, hidden Markov models (HMMs) methods. They search for the enzymatic domains responsible for the different biosynthetic activities in the assembly line process. Analysis of the genes encoded up- and downstream of the hit sequence, then, allows for the identification of whole operons or gene clusters. These websites are quite simple to use. They only require the genome to be submitted, and they produce results regarding the detection and characterisation of secondary metabolites shortly after. AntiSMASH shows the location of the BGCs in the genome, giving a graphical representation and providing further information about the similarity of these detected BGCs with already known compounds. NRPPUR has a very rich database of PKS in particular, and it is a very useful way to detect the NRPS-PKS domains with high accuracy. NAPDOS and ARTS can be very interesting for phylogeny and self-resistance guided antibiotic discovery, respectively. Recently, a new software named gene cluster prediction with conditional random fields (GECCO) [47] showed very high computational performance to identify de novo BGCs. All these pieces of software are, therefore, powerful tools that help to make a selection in silico of the interesting bacteria to be tested in vitro.

The genome mining strategy defined as the “bottom-up approach” [37] is a future-oriented, high-throughput screening approach for bacteria that may be a source of new classes of pharmaceutically active molecules (Table 2). High-throughput screening would enable scientists to detect silent BGCs and to avoid the rediscovery of known metabolites, which is the main cause of the slowdown in search for antibiotics in the pharmaceutical industry [48]. Moreover, the accessibility and ease of use of “bottom-up approach” can help to expand the spectrum of tested bacteria, bringing to light some bacterial genera that were not considered to be important metabolite synthesisers, such as the *Burkholderiales*, *Janthinobacterium*, and *Lysobacter* genera [49]. Thus, the *Lysobacter* genus that has been under exploited for a long time has proven to be very interesting in terms of BGCs for cyclic lipopeptides [50]. The genome analysis of the *Lysobacter* spp. detected an as yet unknown BGC that is only found in one *Lysobacter* species [51]. *Lysobacter antibioticus* ATCC 29479 was cultivated under different conditions to help induce BGC expression. The isolated product was a cyclic lipodepsipeptide NRP called WBP-29479A1, which demonstrated interesting antimicrobial activity against Gram-positive bacteria such as MRSA but less important activity against Gram-negative bacteria and fungi. *Chryseobacterium antibioticum* is a new bacterium isolated from Antarctic soil that exhibits antimicrobial activity against Gram-negative bacteria such as *K. pneumoniae* and *E. coli* bacteria [52]; although the bioactive molecule has not yet been characterised, the genome-based analysis identified three main BGCs of NRPS, PKS, and a hybrid that may be the source of this antimicrobial activity. The in silico prediction of secondary metabolites may facilitate the process of isolation and purification of the metabolite.

Other examples include the genome mining of the newly isolated bacteria using culturomics [53,54,55], which made it possible to culture a large number of as yet unknown species. Genomic analysis of these bacteria from different microbiota showed that they represent a precious source of new antimicrobial compounds. Thus, Fritz et al. (2018) [43], found a *Streptomyces massiliensis* bacterium, which had an antibacterial effect against many Gram-positive and -negative bacteria including MRSA [43]. Likewise, Qin et al. (2017) isolated a new species of filamentous *Actinomycete* bacteria [56] that has been shown to harbour many new and/or atypical BGCs using antiSMASH software. In vitro tests have shown that a strain of *Streptomyces formicae* KY5 demonstrates antagonistic activity against MRSA and against various pathogenic drug-resistant bacteria and fungi. The authors were able to demonstrate that the observed activity was due to a type II PK called formicamycin, using CRISPR/Cas9 gene editing [57]. Genome mining would appear to be an interesting screening method that may help to select potential producing bacteria that deserve to be tested in vitro. This rapid method assists with and prepares the fastidious and time consuming in vitro methods. Moreover, the rapid screening of all bacteria for which the genome is available will help to reinvigorate the research and development of compounds with antimicrobial properties, using these bioinformatics tools.

## 5. Antibiotic Production and Resistance

A successful combat strategy presupposes first and foremost the development of a strong defence. It goes without saying that any bacterium secreting an antibiotic must have in its genome the means to protect itself from the harmful effects of its own weaponry. According to this principle of self-protection against its own antimicrobial metabolites, bacteria should harbour in their genomes the genes encoding for resistance to these antibiotics. Resistance-guided genome mining is an interesting approach, which has led to the identification of BGCs of some already known natural products, and to the discovery of new natural products [58]. Natural antimicrobial substances have different modes of action in order to inhibit growth or induce the death of microorganisms with which the producing bacteria compete in a given environment. These molecules may act by inhibiting DNA replication and transcription, RNA translation, protein synthesis, the proteasome, or the cell wall synthesis. Yet, these target sites of action are consistently present in the antibiotic-producing microorganism, making them vulnerable to the products they have synthesised [59]. With the aim of self-protection, the BGC responsible for antibiotic synthesis usually contains immunity or resistance genes to the synthesised compounds [60]. While trying to find BGCs, it would be consistent to search for a resistance or immunity gene included in a BGC [61]. Furthermore, the mechanism of resistance predicted from the resistance gene can help to characterise the precise mode of action of the potential antibiotic molecule. Thus, Kling et al. (2015) identified in the BGCs encoding for griselimycin, an NRP active against *Mycobacterium tuberculosis* [62], a gene conferring resistance to this anti-tuberculosis compound. This gene, named *griR*, is a homolog of *dnaN* (with 55% protein identity) that encodes for the sliding clamp of DNA polymerase. This work revealed the *dnaN* as an antimicrobial target and helped in evaluating resistance to the modified synthetic griselimycin molecule in order to improve its efficacy and to render it a serious candidate for tuberculosis therapy.

The resistance-guided approach was also used to enrich the antibiotic family of EF-TU inhibitors that were, until then, composed only of four molecules: kirromycin, enacyloxin IIa, pulvomycin, and GE2270A. The EF-TU inhibitors have an activity against Gram-negative bacteria and may represent an alternative to the emergence of resistant Gram-negative bacteria. Yarlagadda et al. (2020) [63] hypothesised that bacteria harbouring the EF-TU resistance gene with the A375T mutation would confer a strong resistance to kirromycin and may be elfamycin producers. When the EF-TU resistance gene sequence was searched against genome databases using the BLAST program, 21 *Streptomyces* sp. were found to harbour homologs to this gene. The search and the characterisation of BGCs using antiSMASH software revealed the presence of these EF-TU resistance genes located inside the synthesis cluster for three *Streptomyces.* One *Streptomyces* bacteria out of the three was found to be a phenelfamycin producer when tested in vitro. Antimicrobial testing showed an interesting activity of this molecule against multidrug resistant gonococci. Although this molecule was already known, this work enabled the identification of a previously unknown elfamycin producer as well as the identification of the BGC of phenelfamycin [63,64]. Other experiments adopting the self-resistance-guided genome mining strategy have also led to the discovery of new antimicrobial compounds. To search for a new antibiotic in the class of topoisomerase inhibitors, Panter et al. (2018) [64] analysed the genomes of an underexploited group of microbes, myxobacteria. This was carried out to look for potential BGCs located next to the pentapeptide repeat proteins, which are responsible for the self-defence mechanism against topoisomerase inhibitors. They succeeded in revealing an as yet unknown BGC, which coded for a new compound called pyxidilicyline, which has an antibacterial and an anticancer effect [64].

## 6. Refined Search for New Variants

Although genomic approaches can identify many potential BGCs, the yield of this method remains unsatisfactory. Indeed, close examination of the predicted NRPs and PKs often reveal many already known antibacterial activities. Many strategies have been developed to try to circumvent the problem of the rediscovery of the same antibiotics. The phylogenetic-guided approach is very useful in order to have primary information on the likely biological functions, structure, and potential mechanism of action, and to understand natural evolution and the diversification of these gene clusters [65]. Based on ketosynthase or condensation domain phylogeny, it seems possible to identify whether the analysed BGC may encode for new chemical entities, by comparison with known domains from known compounds [66]. Several easily accessible pieces of online software, such as NAPDOS [45,65], can detect and extract the elongation domains for NRPS and PKS, the condensation and ketosynthase domains, respectively. This software makes it possible to build a phylogenetic tree from the submitted file by extracting the C and KS domains contained in its database. Results based on the condensation domain’s phylogeny of NRPS give an idea of the aminoacyl binding type. A study analysing soil bacteria made it possible to isolate 50 *Actinomycetes* spp., of which 40 had an antimicrobial activity against blood and food-borne pathogens [67]. Of these, two bacteria, *Micromonospora* Rc5 and *Streptomyces* Ru87, showed interesting bacterial activity in vitro. Phylogenetic analysis of PKS genes suggested that if the metabolites responsible for antimicrobial activity are in the PK family, these would be novel molecules with a unique and previously unknown synthesis pathway. Likewise, based on the phylogeny of KS domain, Guo et al. (2016) identified a new compound with antimicrobial activity, named talafun [68]. Talafun is a PK produced by an endophytic fungus *Talaromyces funiculosus* isolated from a plant, *Silicorna bigelovii.* The phylogenetic analysis placed this PK in the same clade as compounds with unknown biosynthesis pathways. Testing for its antimicrobial activity showed that talafun has better antimicrobial activity than ampicillin, but less than gentamicin against *E. coli.*

Genome databases provide an important source of information to be exploited in the search for new antimicrobial compounds. Targeted searches can be performed to find a particular type of compounds. Thus, Li et al. (2018) [69] analysed as many as 7395 bacterial genomes and targeted cationic nonribosomal peptides (CNRPs). Based on the Stachelhaus code [34], amino acid composition, and residues charges, the authors targeted CNRPs and succeeded in isolating two new compounds: brevicidine and laterocidine. These compounds have bactericidal activities against antibiotic resistant Gram-negative pathogens and colistin resistant *Escherichia coli* [69].

Thaker et al. (2013) [70] applied the phylogeny-driven prediction of new antibiotics to the search for new NRP variants. They looked for glycopeptide antibiotic synthesisers within only vancomycin-resistant bacteria from a library of 1000 *Actinomycetes*. They found 39 resistant bacteria, all of which were positive for amplification of the *vanHAX* resistance genes. Phylogenetic analysis showed that one strain harboured glycopeptide biosynthesis core genes (*oxyB*, *oxyC*, and *dpgC*) that were quite distinct from those involved in the synthesis of vancomycin and teicoplanin. The corresponding metabolite was isolated and named pekiskomycin. This compound showed antimicrobial activity against vancomycin-resistant *Enterococci* type B, although its antibacterial activity is modest. Culp et al. (2020) [71] characterised a new NRP antibiotic called corbomycin by adopting the phylogeny-driven approach. The authors were interested in discovering an antibiotic with a new mechanism of action. To do so, they searched for BGCs that are similar to the known BGCs in terms of glycopeptides’ antibiotic synthesis, but they were excluded from their analysis genomes containing a known self-resistance gene. The phylogeny on the basis of the condensation domain showed that corbomycin is fairly distinct from existing NRPs in the glycopeptide family. The product was isolated and characterised and was found to have a new mode of action by inhibiting autolysin. Carbomycin has demonstrated antimicrobial activity against Gram-positive bacteria in vitro and in vivo against MRSA skin infections in mouse models. This approach gathers data from antibiotic resistance studies together with findings from phylogenetic analysis, and it has been proven to be a fairly effective combination for the detection of new antimicrobial compounds.

## 7. Metagenomic Strategy for Harnessing the Chemical Repertoire in the Environment

Because community composition and functions rely on varying extents of secondary metabolites involved in inter-organism competition and environmental interaction, it is important to estimate and characterise the production of metabolites by organisms in a microbial community. For this purpose, bioinformatic programs have been used to analyse the DNA within an environmental or a microbiota sample, looking for BGCs in the context of metagenomic studies. Metagenomes offer the possibility of collecting a large amount of genetic information on the NRPS-PKS that can be found in a given ecosystem [72,73]. Beyond the constraints of culture, metagenomics allow for the exploration of the potential of yet uncultured, unknown species, giving a more accurate view of the entire reservoir of BGCs in a community [74]. Therefore, the DNA from microbial communities is cloned and expressed in a surrogate host to study the functions of encoded proteins. Identifying BGCs using functional metagenomics consists of screening for enzymatic activities that are involved in metabolite biosynthesis [75]. This functional metagenomic screening led to the discovery of novel enzymes that had not been predicted only from the analysis of genomic sequence of cultured microorganisms [76,77,78].

Hover et al. (2018) discovered malacidin, a new antibiotic that has demonstrated activity against multidrug-resistant Gram-positive pathogens [76]. They, therefore, conducted a sequence-guided screening of samples from various soil reservoir. Then, they searched for a putative calcium-dependent antibiotic by looking for BGCs encoding for the conserved Asp-X-Asp-Gly calcium-binding motif. Likewise, Wu et al. found an antibiotic-dependent calcium called cadaside, using a similar strategy. Cadaside has been shown to be effective against multiple drug-resistant Gram-positive pathogens [77]. Other researchers have gone further, and they have tried to find in this strategy an alternative solution to the problems of antimicrobial resistance. Thus, Peek et al. (2018) [78] assessed the diversity of rifamycin-like gene clusters from 1500 soil samples from different geographical areas [78]. They targeted the universal precursor for the ansamycin family, the 3-amino-5-hydroxy benzoic acid (AHBA) synthase gene using degenerate primers and identified a PK named kanglemycin, which is a rifamycin congener. Kanglemycin showed activity against Gram-positive *Staphylococcus aureus*, *Staphylococcus epidermidis*, and *Listeria monocytogenes* and against clinical isolates of *Mycobacterium tuberculosis*, which are resistant to rifampicin. In summary, metagenomics has revealed a large variety of secondary metabolites with potential antimicrobial activity, including activities against resistant bacteria. The compounds identified with culture methods appear to represent a small and a noticeable part of existing natural metabolites. This is only the tip of the iceberg, as the total number would appear to be really much greater, thanks to community-based analysis using metagenomics. Knowing that antibiotic isolation from soil microbes came to end due to the repetitive rediscovery of existing molecules rather than the discovery of new ones, findings from metagenomics show that it was not a question of material but rather a problem of methodology. Metagenomics turns out to be a very useful complementary method to culture-guided genomics and to genomics in general in order to achieve better sensitivity and more reliability.

## 8. Synthesis of Natural Antibiotics

Secondary metabolites with antimicrobial activity obtained by synthesis from simple molecules are rare compared to products obtained by extraction. Indeed, the particular biosynthesis process of the secondary metabolites, i.e., the assembly of the small monomeric building blocks of amino acids for NRPS and acyl-CoAs for PKS, followed by further modifications by a variety of tailoring enzymes, renders chemical synthesis extremely laborious. The modular nature of NRPS and PKS has inspired the concept of combinatorial biosynthesis to generate unconventional natural products for therapeutic applications. Bioinformatic guiding programs and algorithms, coupled with chemistry, have enabled the development of a new type of antibiotics called synthetic bioinformatic natural products (syn-BNP). The creation of syn-BNPs is very often inspired by the BGCs from bacterial genomes deposited in publicly available databases. Based on the adenylation (with regards to NRPS) or acetylation (with regards to PKS) domain, it is possible to predict the selected substrate and, consequently, the final composition of the molecules encoded by the BGC. This culture-independent approach is dependent upon robust algorithms such as the NRPS predictor [31], Minowa [79], and the Stachelhaus code [30]. Some studies have managed to synthesise molecules based on these predictions and have demonstrated their biological activity [80]. This approach allows for the elaboration of a good matrix for the production of molecules and helps to circumvent the difficulties due to silent BGCs. Moreover, it is no longer necessary to physically possess the strains but rather to work on the genomes available in public databases.

Syn-BNP may, therefore, represent an inexhaustible source of potential new antibiotics [81]. This method has made it possible to identify many interesting molecules in recent years with various mechanisms of action and activity. Chu et al. (2016) screened a large collection of bacterial genomes from the human microbial project database [80]. They were able to synthetise 30 molecules for which they tested their antimicrobial activity against human pathogens. NRPS clusters from *Rhodococcus equi* and *Rhodococcus erythropolis* led to the discovery of the antibiotic humimycin [80]. Humimycin has demonstrated antimicrobial activity against methicillin-resistant *Staphylococcus aureus*, and it has potentiated β-lactam activity. In another work, Chu et al. (2017) selected 96 linear peptides that guided the synthesis of 171 syn-BNPs [81]. Peptides were, then, cyclised, leading to the discovery of nine syn-BNP cyclic peptide antibiotics. All nine compounds showed at least one antimicrobial effect against antibiotic resistant ESKAPE pathogens (*Enterococcus faecium, Staphylococcus aureus, Klebsiella pneumoniae, Acinetobacter baumannii, Pseudomonas aeruginosa*, and *Enterobacter species*). These nine compounds have different mechanisms of action including cell lysis, inhibition cell wall biosynthesis, and membrane depolarisation [81]. A compound known as gladiosyn, the NRPS of which was inspired by a BGC from *Burkholderia gladioli*, demonstrated antimicrobial activity against Gram-positive bacteria but also against most Gram-negative bacteria from the ESKAPE pathogen group when combined with polymyxin. Another syn-BNP named thurinsyn, inspired by the genome of *Bacillus thurigiensis*, has shown a broad spectrum of action, especially antimicrobial activity against *Mycobacterium tuberculosis Hr37*. Two syn-BNPs were considered to be of particular interest, including collimosyn and mucilasyn, which were inspired by the NRP in the genomes of *Collimonas fungivorans* and *Paenibacillus mucilaginosus*, respectively. Collimosyn deregulates the *ClpP* protease and may, therefore, be active against cancer cells. Mucilasyn has shown promising activity against *Acinetobacter baumanii* and has shown no toxicity on human cells in vitro [81].

The results obtained from these studies are very promising with regards to the search for antibiotics. The authors have verified that the synthesised structures do not look like any existing listed natural product. No similar metabolites could be identified previously using classical fermentation methods alone [80]. Thus, this approach opens up a new line of research for antibiotics. Vila-Farres et al. (2017) [82] synthesised a peptide with an antifungal activity inspired by a cluster found in the genome of *Xenorhabdus nematophila*, which could not be detected by culture methods. In the same study, Vila-Farres et al. (2017) synthesised a peptide based on an NRPS found in the genome of *Paenibacillus mucilaginosus* strain K02, which has been shown to be active against Gram-positive bacteria [82]. The synthesised peptide, named paenimucillin A, also showed limited activity against Gram-negative bacteria. Further modifications made by changing the N-acyl at the N-terminal acyl led to the new compound gaining activity against multi-resistant *Acinetobacter baumanii*, while retaining limited toxicity. This new syn-BNP was named paenimucillin C and showed encouraging results in skin wound infections on multidrug-resistant (MDR) *A. baumannii* in a rat model [83]. This approach was revealed to be particularly amenable, while drawing inspiration from known BGCs, and it may also offer the possibility of optimising syn-BNP activity. Given the potential of this method in discovering new antibiotics, the use of classical fermentation or the culture of bacteria did not appear to be relevant. Thus, databases such as NCBI and genome sequencing became a source for the discovery of new antibiotics. Moreover, the laboratory conditions required to generate any experimental resistance against these syn-BNP products did not yield satisfactory results [83]. These findings are very encouraging, because they guarantee that these future pharmaceutical products are effective, secure, and immune to bacterial resistance.

## 9. Dilemma between the Knowledge from In Silico and the Vagaries of In Vitro Methods

A multitude of NRPS-PKS BGCs have been characterised by bioinformatic software, yet it continues to be very tedious in some cases to prove that those clusters result in products with antimicrobial activity. Indeed, some microorganisms with predicted BGCs in their genomes do not show antimicrobial activity in vitro. The difficulty is that we are not sure why this “nonobservation” is occurring. There are two cases in this situation, first the BGC may be expressed, but the product cannot be characterised and remains unknown; second, the BGC is not expressed, and naturally, the product remains unknown and uncharacterised. This situation of known BGC but unknown product [84] is a frustrating one, because the product that would be pharmacologically interesting might never be characterised. Sometimes, culture or molecular techniques can lead to the expression of BGCs that may have potent antimicrobial activity.

Cultivation under different culture conditions may drive the expression and secretion of metabolites. *Streptomyces* sp. KCB13F003 was studied for the first time in search of potential new compounds through LC-MS screening. These investigations led to the discovery of two new cyclic depsipeptides and ulleungamides A and B [85]. *Streptomyces* sp. KCB13F003 genome analysis has revealed multiple putative BGCs, including one NRPS BGC adjacent to the halogenase gene that encodes chlorinated hexapeptides [86]. As this compound was not detected under standard culture conditions, the authors tried different culture media to induce the expression of BGC. They succeeded in isolating two NRP compounds named ulleungmycins A and B. These compounds display an activity against Gram-positive pathogenic bacteria, including quinolone and methicillin-resistant *S. aureus*. More sophisticated methods could achieve this goal, such as heterologous expression and the use of engineered promoter or action on transcript regulators [87]. Thus, *Streptomyces roseosporus*, a well-known microorganism for the synthesis of daptomycin an NRP antibiotic, was found to harbour more than 20 BGCs in its genome [88]. Some of these NRPs, including arylomycins, napsamycins, and stenothricins, were able to be characterised thanks to advances in mass spectrometry and networking analysis [89]. *S. roseosporus* NRRL 15998 harbour a silent BGC type I PKS homolog to the incednine BGC, which was activated by CRISPR-Cas9 technology and led to the discovery of auroramycin [88]. Auroramycin is active against Gram-positive bacteria including MRSA. These examples are a clear illustration of the need for multiple approaches to search for new products.

## 10. Conclusions

The search for new antimicrobial compounds has been neglected by the pharmaceutical industry [90] over the past decade, while antimicrobial resistance in human pathogens has become an issue of increasing concern [91]. The World Health Organization has published a list of human pathogens for which antibiotics are urgently needed [92]. The search for new antibiotics using culture-dependant methods to fight antimicrobial resistance slowed down [90]. Despite the fact that a large variety of NRPS-PKS BGCs exists in the environment around us [93], it is estimated that we only know 10% of the secondary metabolites and less than 1% of the global consortium of microbial producers [94]. Efforts in metagenomic exploration, the discovery of new bacterial species and genome analysis (Figure 2) must be carried out in order to discover new antibiotics representing an alternative solution to available treatment options. Technological advances and a better understanding of NRPS-PKS BGCs have made it possible to implement various approaches to target compounds that may be of interest to researchers. In addition, increasingly powerful analytical tools are available online, as well as databases, which can provide access to a huge amount of genomic data. These in silico approaches facilitate the identification of bacteria to be tested as priority. Furthermore, culture-independent methods [81,82,83] are an interesting way of circumventing difficulties, such as bacteria availability, problems related to culture, and silent BGCs. However, culture under multiple conditions, imitating reality as much as possible, remains a powerful tool for the expression of BGCs. To conclude, the combined use of these methods will expand our knowledge about the NRPS-PKS repertoire and will consequently enable the development of new antibiotics to fight antimicrobial resistance.

## Figures and Tables

**Table 1:** Nonribosomal peptide (NRP) and polyketide (PK) molecules used currently in human medicine.

Synthesis Mode	Class	Antibiotics	Organism	Discovery	Spectrum
RPS	β-Lactams	Penicillin	*Penicillium notatum, Penicillium chrysogenum*	1928	Broad
		Cephalosporin	*Cephalosporium acremonium*	1948	Broad
		Carbapenem	*Streptomyces cattleya*	1976	Broad
		Monobactam	*Chromobacterium violaceum*	1981	Aerobic Gram-negative bacilli
	Glycopeptides	Vancomycin Teicoplanin	*Amycolatopsis orientalis*	1953	Gram-positive
			*Actinoplanes teichomyceticus*	1978	Gram-positive
	Polypeptides	Polymyxin	*Paenibacillus polymyxa*	1947	Gram-negative
	Streptogramins	Streptogramin B	*Streptomyces graminofaciens*	1953	Gram-positive
		Pristinamycin	*Streptomyces pristinaespiralis*	1961	Gram-positive
	Lincosamides	Lincomycin	*Streptomyces lincolnensis*	1963	Gram-positive and some anaerobic bacteria
	Lipopeptides	Daptomycin	*Streptomyces roseosporus*	1986	Gram-positive
PKS	Macrolides	Erythromycin	*Streptomyces erythraeus*	1948	Broad
		Josamycin	*Streptomyces narbonensis var. josamyceticus*	1967	Broad
		Midecamycin	*Streptomyces mycarofaciens*	1975	Broad
		Spiramycin	*Streptomyces ambofaciens*	1952	Broad
		Fidaxomicin	*Dactylosporangium aurantiacum subsp. hamdenesis*	1975	Broad
	Tetracyclines	Chlortetracycline	*Streptomyces aureofaciens, Streptomyces rimosus*	1948	Broad
	Carboxylic acid	Mupirocin	*Pseudomonas fluorescens*	1971	Aerobic Gram-positive and negative
Hybrid NRPS/PKS	Rifamycins	Rifampicin	*Streptomyces mediterranei*	1957	Broad

**Table 2:** New Nonribosomal peptide (NRP) and polyketide (PK) products discovered by foreign in silico/in vitro strategies.

Discovery Approach		Authors Target	Antibiotics	Activity	Synthesis
Culture-dependant first discoveries	Ecology guided		Lugdunin	*S. aureus (Mrsa, GISA), Enterococcus (VRE) *	NRPS
			Teixobactin	Gram-positive bacteria, *Mycobacterium tuberculosis*	NRPS
*In silico/In vitro*	Classical screen		Formicamycins	*Staphylococcus aureus* (MRSA), various pathogenic drug resistant bacteria and fungi	PKS
			WBP-29479A1	Gram positive bacteria including MRSA	NRPS
	Self-resistance guided	Topoisomerase inhibitors	Pyxidilicyline	Antibacterial and anticancerous effect	PKS
	Specific interaction modes	Cationic nonribosomal peptides	Brevicidine	Antibiotic resistant gram-negative pathogen and colistin resistant *Escherichia coli*	NRPS
		Cationic nonribosomal peptides	Laterocidine	Antibiotic resistant gram-negative pathogen and colistin resistant *Escherichia coli*	NRPS
		Calcium-dependent antibiotic	Malacidin	Multiple drug-resistant Gram-positive pathogens	NRPS
		Calcium-dependent antibiotic	Cadasides	Multiple drug-resistant Gram-positive pathogens	NRPS
	Phylogeny guided		Talafun	*Escherichia coli*	PKS
		Glycopeptide related with new mode of action	Carbomycin	Gram+ bacteria, *Staphylococcus aureus* (MRSA).	NRPS
	Silent BGC:diverse culture condition		Ulleungmycins A and B	Gram +, quinolone-resistant and methicillin-resistant *Staphylococcus aureus*.	NRPS
	Silent BGC:Crisp cas9 activation		Auroramycin	Gram-positive bacteria including methicillin resistant *Staphylococcus aureus* (MRSA)	PKS
	Syn-BNP		Humimycin	Gram +	NRPS
			Paenimucillin A	Gram +/−	NRPS
			Paenimucillin C	Gram - *Acinetobacter baumanii*	NRPS
			Gladiosyn	Gram +/−	NRPS
			Thurinsyn	Gram +/− *Mycobacterium tuberculosis*	NRPS
			Mucilasyn	Gram +/− *Acinetobacter baumanii*	NRPS

## Data Availability

Not applicable.
